# Metabolism of myeloid cells in brain tumors

**DOI:** 10.3389/fimmu.2026.1894833

**Published:** 2026-07-14

**Authors:** Aashna Saxena, Defne Bayik

**Affiliations:** 1 Department of Molecular and Cellular Pharmacology, University of Miami, Miami, FL, United States; 2 Sylvester Comprehensive Cancer Center, University of Miami, Miami, FL, United States

**Keywords:** brain tumor, glioblastoma, immunometabolism, macrophage, MDSC, metabolism, microglia, brain metastasis

## Abstract

Primary and recurrent brain tumors are aggressive malignancies with high mortality rates and limited treatment options. Glioblastoma (GBM) in particular are largely refractory to immunotherapies despite harboring a significant proportion of immune cells in the tumor microenvironment (TME). Increasing evidence suggests that the immunosuppressive TME in brain tumors is driven by functional and metabolic reprogramming of resident and infiltrating myeloid cells. Here, we examine the determinants of immunometabolic landscape in brain tumors and distinct features of heterogeneous myeloid cell populations. We discuss how interactions between cell types and metabolic programs shape spatial niches. We further dissect the effect of tumor stage, type and therapy in informing metabolic features of the TME. Collectively, this systematic review provides an overview of myeloid cell metabolism in brain tumors and highlights potential opportunities for future studies targeting metabolic states for cancer immunotherapy.

## Introduction

The brain was long considered an immune-privileged organ, that restricts the access of immune cells to restrain detrimental effects of inflammation via an impermeable blood-brain barrier. However, this view was challenged by the identification of meningeal lymphatics and the detection of large number of immune cells in neurological diseases (1–3). Research has since revealed controlled interactions with peripheral immune cells in the meninges, choroid plexus, and perivascular spaces along with the brain resident microglia under homeostatic conditions, which become altered in the context of diseases - including in brain tumors (4).

Glioblastoma (GBM), the most common primary malignant brain tumor, is a highly aggressive cancer with a 5-year survival rate of roughly 5%. Current standard of care treatment of surgery, radiation and chemotherapy often fails (5, 6). Despite considered immunologically “cold”, due to a low tumor mutational burden, immune cells are abundant in the GBM tumor microenvironment (TME). Tumor-associated resident and infiltrating myeloid cells can constitute up to 30% of tumor mass, suggesting that they play an important role in disease progression (7). The myeloid component of the TME consists of bone marrow-derived macrophages (BMDM), tumor-associated neutrophils (TANs), and myeloid-derived suppressor cells (MDSCs) and yolk sac-derived resident microglia (8). While functional roles of these myeloid cells are well-established, there is limited knowledge on the molecular determinants of heterogenous and dynamic myeloid cell responses. Recent studies suggest that myeloid cells are differentially shaped by the hypoxic and acidic GBM TME, which comprises a milieu of cytokines, chemokines, metabolites and other factors secreted by cancer and stromal cells (9, 10). These factors collectively lead to the metabolic rewiring and functional reprogramming of individual immune cells, further driving immunosuppression and propagating tumor supportive functions. The metabolism of resident or infiltrating myeloid cells in the GBM TME was shown to substantially differ from those in normal brain or blood from patients with brain tumors (11). For example, under physiological conditions, activated microglia maintain surveillance functions and guide neural circuitry through oxidative metabolism and elongated cellular processes (12). Within the GBM TME, however, microglia lose inflammatory phenotypes and undergo metabolic adaptation. Tumor-associated microglia exhibit altered lipid metabolism, including increased APOC1 expression compared to normal brain microglia (13). Additionally, they adapt to the nutrient-deprived TME, where most cell types compete for glucose. GBM-associated microglia uniquely utilize other metabolites, such as fructose, via upregulation of the fructose transporter GLUT5. Fructose metabolism via the polyol pathway not only sustains microglia, but also generates NADH and buffers reactive oxygen species (ROS), limiting inflammatory signaling and supporting an immunosuppressive phenotype (14). Similarly, tumor-associated MDSCs are characterized by distinct lipid and carbohydrate metabolism that was not observed in peripheral populations, further emphasizing that the GBM TME actively reprograms immune metabolism (11). Based on this emerging evidence, the current review discusses the role of key factors in mediating tumor–immune metabolic crosstalk, and the implications of immunometabolic reprogramming in brain tumor heterogeneity, treatment resistance, and therapeutic targeting.

## Spatially distinct immunometabolism

GBM are characterized by extensive spatial and cellular heterogeneity, with distinct microenvironmental niches shaping both cancer cell identity and immune cell function. Transcriptional profiling of glioma cells has identified four unique states - neural progenitor-like (NPC), oligodendrocyte progenitor-like (OPC), astrocytic (AC), and mesenchymal (MES), which lead to inter- and intratumoral heterogeneity (15). Each of these cell states is preferentially abundant in specific regions of the tumor, such as the hypoxic/necrotic core, pseudopalisading necrosis (PAN), microvascular proliferation (MVP) zone, and the leading edge. MES-like glioma cells, which are associated with poor prognosis, therapy resistance, and recurrence (16), are typically found in the core and PAN regions, both of which are hypoxic. In contrast, NPC- and OPC-like cells are more commonly found in perivascular regions (17). While this classification is based on neural cell types, another approach using transcriptional data categorized glioma based on biological pathways, into a distinct set of four states – glycolytic/plurimetabolic (GPM), mitochondrial (MTC), neuronal (NEU) and proliferative/progenitor (PPR). Comparing this system with the neural/developmental classification revealed similarities between OPC and PPR, NPC and NEU, AC and MTC, and MES and GPM cellular states and their localization (18). Metabolic features of the MES/GPM subtype, such as increased glycolysis, as well as increased metabolism of lipids, amino acids, iron and sulfur is indicative of the adaptation of glioma cells to their micro-niches and highlights the spatial regulation of cellular phenotypes.

**Figure 1:**
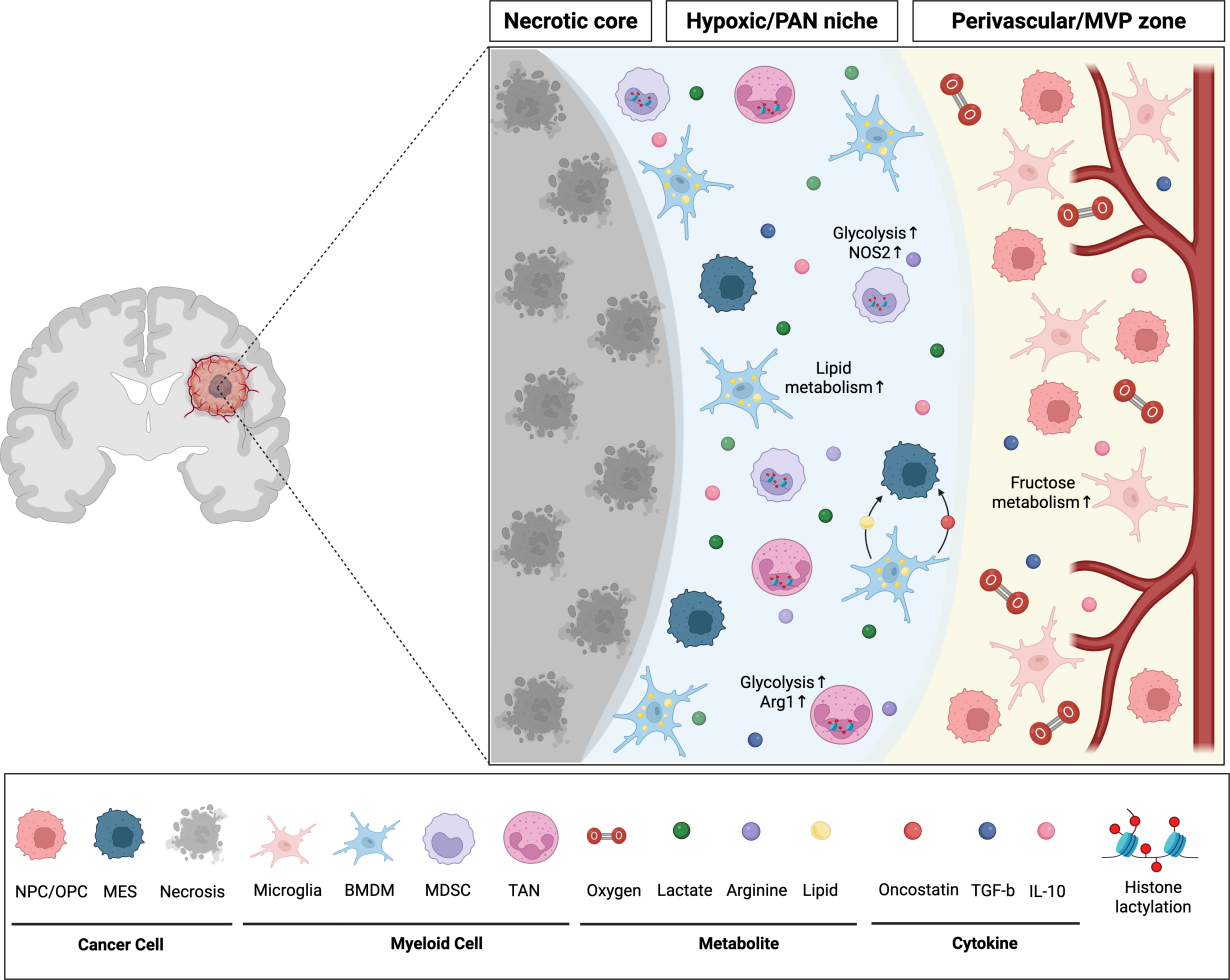
Spatial organization and immunometabolism of the GBM TME. Created with BioRender.com.

Emerging studies highlight that distinct immune populations also localize to specific tumor niches, which reinforce regional metabolic programs (**Figure 1**). Microglia are typically enriched in vascular and peritumoral regions, although they can also localize within tumor cores, where they engage in oxidative and lipid metabolism (19, 20). In contrast, BMDMs, TANs, and MDSCs are more prominent in hypoxic regions, highlighting a site-specific localization pattern for resident vs infiltrating myeloid populations (21). This niche has reduced infiltration of lymphocytes such as NK cells and CD4+, and CD8+ T cells as a result of both reduced vasculature as well as the myeloid cell-mediated suppression (22). Importantly, these niches are critical to establish the metabolic-functional properties of immune cells. MDSCs and TANs at the hypoxic regions are characterized by high glycolysis, which has been shown to be critical for mitigating ROS damage in these cells (23). Lactate accumulation via glycolysis in both glioma and MDSCs can directly regulate gene expression through histone lactylation as well. H3H4 lysine lactylation (Kla) increases chromatin accessibility, epigenetically promoting the expression of immunosuppressive mediators such as Arginase-1 (Arg1), and nitric oxide synthase 2 (NOS2) and drive secretion of cytokines such as TGF-b, and IL-10 that recruit additional suppressive immune populations into the TME (24, 25). Arg1 and NOS2 are the two main drivers of MDSC-mediated metabolic immunosuppression, which work by utilizing arginine as a substrate, thus depleting its pool in the TME. This reduction has a suppressive effect on CD8+ T cells, that require arginine for cell cycle progression (26) and expression of the TCR CD3zeta chain (27). Arg1 is an enzyme that breaks down arginine to urea and ornithine (28), while NOS2 converts it to nitric oxide (NO) and citrulline (29). Besides depriving cytotoxic T cells of arginine, these enzymes induce immunosuppression via secondary metabolic changes as well. Ornithine produced by Arg1 is a precursor for polyamines, which are produced in MDSCs as a buffer against the acidic TME (10). NO stabilizes hypoxia-inducible factor (HIF1a) resulting in increased hypoxic metabolism such as glycolysis (30). Citrulline can be used to regenerate arginine, feed into the TCA cycle, or even be used directly for post-translational modifications (PTMs), with histone citrullination regulating TAN phenotype (31, 32). NO further recruits and activates suppressive myeloid populations and regulatory T cells (33). Collectively, these studies indicate that myeloid cells are not passive bystanders and they contribute to the shaping of the metabolic architecture of the niches.

Importantly, niche specific metabolic and subsequent signaling effects on immune cells are informed by the lineage identity. In the hypoxic niche, macrophages and microglia phagocytose myelin debris and become lipid-laden macrophages capable of recycling cholesterol and other lipids to glioma cells (34). This lipid transfer promotes tumor survival under nutrient stress and drives transition toward MES states (34). MES-associated macrophages can also induce mesenchymal transition in glioma cells through secretion of factors such as oncostatin-M, while being reprogrammed by glioma cells to acquire MES-like transcriptional programs, including expression of vimentin, CD44 and annexin-A1 (35). CD44 expression in macrophages is associated with an immunosuppressive phenotype in gliomas (36), while annexin-A1 promotes anti-inflammatory, reparative macrophage signaling in muscle injury (37). Vimentin has varied roles across disease contexts; it promotes extracellular matrix (ECM) degradation in lung cancer but is associated with pro-inflammatory macrophage activation in coronary artery disease (38, 39). Moreover, vimentin affects metabolism of macrophages as well, suppressing ROS production and supporting lipid uptake (40, 41), pointing a complex interaction between cancer-myeloid cell metabolic and functional crosstalk.

The advent of single-cell technologies has supported various efforts to further characterize the spatiofunctional relationship in TME, looking at the niches, cell lineage and functional phenotypes. In fact, a recent study suggested that myeloid cells exist along a plastic-dynamic continuum in GBM and their state depends on TME cues. They identified 4 functional myeloid programs - microglial inflammatory, systemic inflammatory, scavenger immunosuppressive and complement suppressive states. These states localize in different tumor regions, with the hypoxic niche enriched for scavenger and complement suppressive cell types, while vascular niches contained complement suppressive and microglial inflammatory myeloid cells. Comparison by ontogeny revealed that most macrophages were suppressive scavengers, while microglia adopted inflammatory states that were excluded from the hypoxic niche. Importantly, the scavenger and microglial programs were unique to brain tumors, indicating that the brain TME imposes tissue-specific immunometabolic states (42). While these studies highlight the importance of TME in shaping the local immune responses, tumor origin, stage, mutational status and therapy exposure can also provide high-level cues to define immunometabolic states.

## High-grade and low-grade glioma

The World Health Organization categorized glioma into four stages based on histopathological features, with grades 1, and 2 constituting low-grade glioma (LGG) and grades 3, and 4 comprising high-grade gliomas (HGG) (43). LGG can progress into HGG but generally have a better prognosis. Isocitrate dehydrogenase 1/2 (IDH1/2) mutations, one of the common mutations in LGG, found in over 80% of cases (44). Mutations in this gene are rare in HGG, but their presence is correlated with a prolonged survival as well (45). IDH is a key enzyme in the TCA cycle that converts isocitrate into α-ketoglutarate (α-KG) (46). However, mutant IDH (IDHm) converts α-KG into D-2-hydroxyglutarate (D-2-HG), which directly inhibits the TET family of DNA demethylases and Jumonji family of histone demethylases, resulting in broad hypermethylation. These changes occur both in cancer cells, where the D-2-HG is produced, and in immune cells that internalize secreted D-2-HG (47, 48). As a result, IDHm tumors have significantly different metabolic and transcriptional profiles, that inform survival outcomes, and treatment responses.

A consequence of IDHm-mediated epigenetic programming is the repression of lactate dehydrogenase (LDH), the enzyme that converts pyruvate into lactate, and reduces ROS by regenerating nicotinamide adenine dinucleotide (NAD) (47). Thus, D-2-HG is the primary oncometabolite in the IDHm TME, while lactate is the primary oncometabolite in wild-type IDH (IDHwt) gliomas. This dichotomy broadly impacts the TME, leading to distinct immune cell metabolism and composition between the tumor types. IDHm gliomas are enriched for resident microglia and granulocytic/polymorphonuclear MDSCs (G-MDSCs), while displaying reduced infiltration of BMDMs and monocytic MDSCs (M-MDSCs) (49). While both MDSCs suppress immune response by regulating arginine metabolism, they do so via different downstream mechanisms. G-MDSCs primarily express Arg1, while M-MDSCs use NOS2 to suppress T cells (50). G-MDSCs within the IDHm TME appear less immunosuppressive than those in IDHwt gliomas, exhibiting reduced expression of Arg1, due to enhanced G-CSF secretion from IDHm gliomas as a result of epigenetic reprogramming (51). The fraction and phenotype of M-MDSCs, however, is not affected by this. Interestingly, studies in lung and colon cancer have identified that MDSCs respond to G-CSF and GM-CSF by increasing lipid uptake, fatty acid oxidation and immunosuppression, characterized by both Arg1 and NOS2 upregulation (52). These conflicting responses to G-CSF may be driven by tumor genetic background and unique metabolite availability as a result of IDHm gliomas having high D-2-HG and low αKG levels compared to lung/colon cancer. This variability also highlights the potential role of organ-specific effects on infiltrating immune cells. While the metabolic-signaling interplay that leads to a reduction in MDSC suppressive phenotype remains to be studied, IDHm tumors still maintain an immunosuppressive microenvironment through alternative mechanisms. These tumors produce lower levels of chemokines CXCL9 and CXCL10, limiting infiltration of cytotoxic CD8+ T cells (53). In parallel, D-2-HG directly reprograms glioma-associated macrophages by activating tryptophan-2,3-dioxygenase (TDO), an enzyme involved in the catabolism of tryptophan (54). The resulting catabolite, kynurenine, acts as an activating ligand for aryl hydrocarbon receptor (AHR), which creates an immunosuppressive environment by increasing TGFβ signaling and IL-10 production from these macrophages (55). D-2-HG also directly induces extensive epigenetic remodeling within immune cells. Microglia exhibit widespread hypermethylation in IDHm tumors relative to both IDHwt gliomas and non-tumor brain tissue (56). Hypermethylated loci include genes regulating lineage identity, inflammatory activation, chemotaxis, and glycolysis (56), suggesting that secreted D-2-HG drives metabolic rewiring of microglia toward oxidative phosphorylation, and prevents inflammatory signaling.

In contrast, IDHwt gliomas are dominated by a highly glycolytic and acidic microenvironment driven by lactate accumulation. These tumors exhibit increased infiltration of BMDMs, M-MDSCs, some G-MDSCs, and additional suppressive myeloid populations (49). Recent single-cell analyses identified three myeloid populations in IDHwt tumors: early-stage MDSCs (E-MDSCs), M-MDSCs, and macrophages, which display overlapping inflammatory-suppressive transcriptional programs. E-MDSCs and M-MDSCs undergo extensive metabolic rewiring compared to peripheral blood MDSCs of patients with glioma, including increased glycolysis, fatty acid metabolism, fructose and mannose metabolism, pentose phosphate pathway activity, amino acid metabolism, and oxidative phosphorylation-associated gene expression (11). E-MDSCs exhibit particularly high glycolytic activity and preferentially localize to PAN regions, which are characterized by presence of glioma stem cells (GSCs), hypoxia, and aberrant microvasculature, whereas M-MDSCs localize outside these niches. Within these regions, E-MDSCs and GSCs engage in bidirectional interactions whereby E-MDSCs promote cancer growth and immune evasion, while GSCs support MDSC accumulation and proliferation (11). Importantly, lactate can have a direct role in this interaction, as lactate reinforces suppressive myeloid phenotypes by stabilizing HIF1a in MDSCs (57), which induces expression of Arg1 and NOS2. Acidification of the TME due to lactate accumulation also suppresses NK cell activity, induces CD8+ T cell anergy, promotes TAN survival, and impairs mitochondrial respiration in T cells (58). Thus, lactate drives immune dysfunction through a multitude of mechanisms, including epigenetic and indirect metabolic regulation in IDHwt gliomas, likely contributing to disease outcomes.

IDH status represents only one example of how tumor genotypes shape the TME. Other oncogenic alterations may also influence immune composition and metabolic state indirectly. NF1 loss, a major driver mutation linked to the MES subtype, has been associated with increased macrophage recruitment (22). Loss of NF1 leads to accumulation of the metabolites lactate and succinate in the TME, via Ras/ERK signaling that increases glycolysis and inhibits succinate dehydrogenase in the TCA cycle (59). These increase the hypoxic response, which recruits macrophages via secreted ligands VEGF and endothelin-2 (60, 61). Additionally, lactate and succinate support immunosuppressive macrophage polarization (62). EGFR alterations are exclude tumor-associated monocytes that produce EGFR ligands such as epiregulin (EREG) and amphiregulin (AREG), which promote mesenchymal tumor progression (63). In addition, numerous metabolites beyond D-2-HG and lactate, including mannose, glutamate, creatine, kynurenine, succinate, cholesterol-derived lipids, and arginine metabolites, further shape immune cell function and therapeutic response within glioma (64) (**Table 1**).

**Table 1:** Common oncometabolites in the TME.

Metabolite	Source	Effect on TME	Citation
Lactate	Glycolysis product pyruvate is converted into lactate via LDH; produced by multiple cell types in the glioma TME	Increased MDSC suppression, TAN survival, M2 polarization, CD8+ T cell exhaustion	(23–25, 58, 65)
D-2-HG	Mutated IDH converts isocitrate into D-2-HG in glioma cells	Secretion of IL-10 and TGFb by macrophages, increased OXPHOS in microglia	(47, 48, 54–56)
Kynurenine	Tryptophan metabolism via TDO in glioma and immune cells	Secretion of immunosuppressive cytokines TGFb and IL-10 from macrophages	(54, 55, 66)
Adenosine	Breakdown of extracellular ATP via CD39/CD73 expressed in multiple TME cell components	Adenosine signaling mediated release of IL-10, VEGF and TGFb from macrophages, MDSCs and Tregs, suppression of CD8+ T cells	(67–71)
Fructose	Present in high concentration in CNS	Supports microglial survival, suppresses inflammatory signaling	(14, 72)
Succinate	TCA cycle intermediate	Promote M2-like polarization of macrophages	(59, 62)
Creatine	Produced by arginine metabolism in myeloid cells	Protects glioma cells from hypoxic and metabolic stress	(73)
Glutamate	Most abundant excitatory neurotransmitter in the brain	Suppressed T cell proliferation and activation	(74, 75)
GABA	Most abundant inhibitory neurotransmitter in the brain	Increased Arg1 and NOS2 expression in G-MDSCs	(76, 77)
Quinolinic acid	Tryptophan degradation in microglia	Protects glioma cells from redox stress	(78, 79)
Prostaglandin E2	Derived from arachidonic acid via COX2, high production by MES glioma	Immune suppression, secretion of IL-10 by immune cells and induction of angiogenesis	(80–84)
Branched-chain ketoacids	Branched-chain amino acid metabolism in glioma cells	Reduced phagocytic activity of macrophages	(85)

## Brain metastases

Brain metastases exhibit a distinct immune and metabolic landscape compared to primary brain tumors. Unlike GBM, which is predominantly myeloid cell-enriched, brain metastases consist of a higher abundance of lymphoid cells, including both CD4+ and CD8+ T cells, with the main infiltrating myeloid cell type being TANs, recruited via microglia-secreted CXCL8 (49). Despite the presence of infiltrating lymphocytes and partial responsiveness to immune checkpoint inhibitors (ICIs), brain metastases remain more immunosuppressive than primary tumors of origin (86), due to brain-specific constraints such as the blood-brain barrier, local nutrient availability, and the suppressive roles of brain-resident cell types (86). The immune composition also depends on the tumor origin, with melanoma brain metastases having more T cells, while breast cancer brain metastases are more enriched in TANs (49). Brain metastases are thus influenced by both tissue-of-origin as well as brain-specific environmental cues.

Spatial organization of immune cells differs between brain metastases and primary gliomas. In gliomas, microglia may be within the tumor core, while in brain metastases, microglia are more frequently localized to the tumor periphery which may favor interactions with vasculature and stromal components rather than direct interaction with the cancer cells (19). Functions of infiltrating immune cells are also altered, with TANs exhibiting lower reduced ROS production and increased survival than peripheral neutrophils (87). Additionally, they display a more pro-inflammatory phenotype, characterized by enhanced TNF signaling. TANs from brain metastases also had higher TNFa and mTORC signaling when compared to those from glioma (87). While how primary versus secondary cancers differentially shape the immune responses remains to be investigated, studies have shown that lung and breast cancer cells can reprogram astrocytes via exosomes. Cancer-derived exosomes alter astrocytic glucose, amino acid and fatty acid metabolisms, creating a more nutrient-rich environment for seeding (88). Since astrocytes also recruit immunosuppressive macrophages via CCL2 and support cancer cell growth and metabolism by transfer of cholesterol in primary gliomas (89), there are multiple metabolic drivers of cancer-astrocyte-immune crosstalk which could be conserved or divergent between tumor types. An additional factor to consider is the unique enrichment of neurotransmitters in the brain TME, especially since astrocytes are the main regulators of neurotransmitter metabolism. Studies have shown that neurotransmitters can interact with cancer as well as immune cells. In GBM, GABA signaling mediated via GABA receptor B in G-MDSCs increases arginine metabolism and expression of NOS2, making them more immunosuppressive (77). While it remains to be analyzed how neurotransmitter metabolism interacts with broader suppressive networks and metabolic pathways, research has shown that breast cancer cells that metastasize to the brain upregulate GABA receptor expression and signaling (90).

## Therapy resistance

Therapeutic interventions impact not only cancer cells but also the metabolic landscape of the TME, with profound consequences on immune function. While direct studies in GBM are limited, emerging evidence from studies on brain tumors and other solid cancers suggest that treatment-associated metabolic and immune reprogramming can be a major driver of resistance to multiple forms of therapy. Standard-of-care approaches for brain tumors include chemotherapy and radiotherapy, which significantly impacts cellular redox metabolism and immune cell infiltration/function (91, 92).

A patient cohort study comparing newly diagnosed and recurrent GBM demonstrated that microglia predominated in newly diagnosed tumors, while BMDMs were enriched in recurrent post-therapy tumors (93). Importantly, these macrophages exhibited diverse transcriptional and metabolic states, including transitory, phagocytic/lipid metabolism-associated, hypoxic, microglial-like, interferon-responsive, and non-microglial states (93). Although many of these states were present in both newly diagnosed and recurrent tumors, recurrent GBM showed marked enrichment of hypoxic and non-microglial macrophage populations, suggesting that treatment can inform immunometabolic features of TME (93). Radiation induces hypoxia and oxidative stress within brain tumors, and studies have shown that patients with shorter relapse intervals following radiotherapy exhibited increased macrophage abundance within the TME (22). Studies in breast cancer provide mechanistic insight into how these processes may occur. Radiation-induced tissue damage increases HIF1a signaling (94), resulting in enhanced angiogenesis and accumulation of MDSCs (95). This also promotes glycolysis and lactate production while simultaneously increasing expression of Arg1 and PD-L1 in myeloid cells, driving them toward more immunosuppressive phenotypes (96). In addition to increased immunosuppression, hypoxia also directly drives radioresistance, since oxygen is required for radiation-driven cell death (97). Given that brain tumors, both metastases and gliomas, are treated with radiotherapy and already contain extensive hypoxic niches, treatment may further amplify these conditions. A higher dose of radiotherapy may be needed to overcome this, which could lead to toxicity. To avoid these detrimental side effects, stereotactic radiosurgery, focused radiation on the tumor, has replaced whole brain radiotherapy, though metabolic effects remain to be studied (98).

Lipid metabolism has also emerged as a regulator of therapy resistance, with radiation increasing lipid metabolism and accumulation within glioma cells to protect them from endoplasmic reticulum stress and apoptosis (99, 100). This also impact the immune environment, since excess lipids increase production of immunosuppressive prostaglandin E2. In the model of radioresistance, lipid accumulation in cancer cells was driven by increased fatty acid synthase (100). However, transfer of lipids from lipid-laden macrophages to cancer cells has been shown to sustain GBM growth and may contribute to radioresistance as well (99). Aberrant lipid metabolism also drives resistance to PD-1 blockade immunotherapy in lung cancer, resulting in T cell dysfunction and apoptosis. In GBM, poor response to PD-1 blockade was associated with increased scavenger-like myeloid cells (42).

In addition to treatments that target the tumor and the TME, patients also receive palliative therapy. Edema, a common symptom of brain tumors caused by the disrupted vasculature, is typically managed with glucocorticoids such as dexamethasone (101). However, this can have several adverse effects including immunosuppression (102). Characterization of myeloid cells in the TME of patients revealed an increase in a suppressive phenotype among patients treated with dexamethasone (42). This effect is mediated via glucocorticoid-induced reprogramming of mitochondrial metabolism in macrophages, with increased TCA cycle activity and production of metabolite itaconate, that disrupts the expression of pro-inflammatory genes (103). These findings reinforce the concept of bidirectional metabolic crosstalk between cancer and immune cells, where therapy-induced metabolic adaptations simultaneously support tumor fitness and immune suppression, and may mediate resistance to more than one form of therapy. Thus, an efficient therapeutic approach must consider targeting the unique metabolic needs of the TME concomitantly with radiation, chemotherapy, and immunotherapy. These therapeutic avenues may differ by tumor type, stage as well as previous therapeutic exposure. For instance, blocking the synthesis of D-2-HG via targeted inhibitors of IDH such as Vorasidenib may improve LGG outcomes (104). Inhibiting oncometabolites has proven effective in reprogramming immune populations – inhibiting lactate production in HGG led to reduced suppressive phenotypes of TANs via reduced histone lactylation, resulting in prolonged survival (24). In lung cancer, radiation was shown to recruit and activate macrophages via secretion of cytokines CSF1 and CCL2. However, blocking CD47-driven immune-evasive macrophage signaling in combination with radiation led to improved outcomes, including abscopal effects (105). Rewiring immune cell metabolism (for example arginine metabolism in MDSCs and lipid metabolism in macrophages), as well as depleting myeloid cell populations via anti-CSF1R/anti-CCL2 strategies can inform immunosuppressive states in brain tumors. Another potential strategy for treatment of brain tumors could involve modulating hypoxia. This is used to treat a form of renal cancer that is characterized and driven by hypoxia, using inhibitors against HIF signaling (106, 107). In line with this notion the limited effect of the anti-VEGF antibody, bevacizumab, in GBM could be linked to increased hypoxia, resulting in aggressive tumor growth (108). Ultimately, a combinatorial approach of these treatments can improve response to standard of care as well as decrease immunosuppressive metabolic programs.

## Discussion

While dysregulation of metabolism in cancer cells is an established hallmark of cancer, similar dysregulation in other cellular components of the TME is emerging as a defining feature of malignancy (109). Increasing evidence demonstrates that distinct mutational backgrounds generate unique immune phenotypes across brain tumors, including brain metastases, IDHm LGG gliomas and IDHwt HGG gliomas, by establishing metabolically distinct ecosystems that dictate immune cell recruitment, metabolism and function.

Immunometabolic rewiring orchestrates immunosuppression through multiple mechanisms. First, tumor-derived metabolites can directly induce changes in both cancer and immune cells, via epigenetic modifications, or downstream signaling (56, 110). These changes may increase recruitment of suppressive cells via cytokine release, promote fitness and suppressive phenotypes of immune cells. Second, tumor cells and several immunosuppressive myeloid cells compete with cytotoxic T cells for key nutrients like glucose and arginine, leading to exhaustion, which is exacerbated by the hostile, acidic TME created by release of metabolites such as lactate into the extracellular space (27, 58). Besides immunosuppression, metabolic reprogramming in immune cells also directly facilitates tumor growth and progression by crosstalk and metabolite transfer with cancer cells (99). In addition to these mechanisms are the factors unique to the brain that must be considered. The blood-brain barrier imposes restrictions on proliferation by limiting nutrient availability, forcing both cancer and immune cells to metabolically adapt (111). Additionally, glial cells apart from resident immune microglia, such as astrocytes and oligodendrocytes interact within the TME (89). Neurotransmitters are another distinctive feature of the brain TME; glutamate and GABA are the most abundant excitatory and inhibitory neurotransmitters in the brain respectively, and their roles in immune modulation and metabolism are being uncovered (74–76).

Moreover, confounding factors for immunometabolic reprogramming exist at the systemic level. For instances, there are sex differences in immunity, wherein females generally have a stronger immune response than males but are at higher risk of autoimmune conditions and chronic inflammation, while males are susceptible to infections and cancer (112). GBM itself is a sexually dimorphic disease, with higher incidence and worse prognosis in males than in females (113). In the context of immunometabolic reprogramming, research has revealed female-specific metabolic rewiring in certain immune populations in the GBM TME, driven by neurotransmitter signaling, while recent studies highlight that androgen signaling interacts with glucocorticoid metabolism to establish immune states in males (77, 114). Age also represents a major determinant of immunometabolism. Inflammaging presents as chronic activation of innate, myeloid immune cells, and impaired adaptive immune functions, a phenotype that is linked to cancer incidence in older individuals (115). While there are limited studies on the link between immunometabolism in aging and cancer, studies have shown that altered immune metabolism impacts inflammaging and vice versa, in viral infections (116). Environmental and lifestyle factors such as diet, obesity and microbiome also significantly influence immune cell metabolism and function. Nutrient availability and diet modulate metabolism of carbohydrates, lipids and amino acids in both tumor and immune cells (117). In fact, several dietary interventions (such as ketogenic diet) have been investigated for their ability to modulate glioma metabolism and immune response (118). Thus, despite the perception of the brain as an organ isolated from several external factors due to the restrictive blood-brain barrier, brain tumors and their immune metabolism cannot be studied in isolation from systemic host metabolism and environmental influences.

Understanding immunometabolic reprogramming across different brain tumor types and patient populations is essential to improve clinical outcomes; characterization of tumor-specific metabolic signatures may lead to development of biomarkers for disease prognosis, therapy response and resistance. Further, targeting unique metabolic dependencies of tumor and pro-tumor immune populations in combination with standard therapy may improve outcomes. Considering the extensive inter-individual and intratumoral heterogeneity, therapeutic approaches should involve personalized, combinatorial and dynamic strategies that can adapt to the complex and evolving tumor and immune metabolic landscape of brain tumors.
